# Dual anti-HSV and anti-HIV activity of the lantibiotic Labyrinthopeptin A1

**DOI:** 10.1186/1471-2334-14-S2-P79

**Published:** 2014-05-23

**Authors:** Geoffrey Férir, Mariya I Petrova, Graciela Andrei, Robert Snoeck, Mark Brönstrup, Roderich D Süssmuth, Dominique Schols

**Affiliations:** 1 Rega Institute for Medical Research, University of Leuven, Leuven, Belgium; 2 Center of Microbial and Plant Genetics, University of Leuven, Leuven, Belgium; 3 Helmholtz Center for Infection Research, Braunschweig, Germany; 4 Technische Universität Berlin, Fakultät II – Institut für Chemie, Berlin, Germany

## Background

It has been shown that genital lesions and altered innate mucosal immunity caused by HSV-2 are important cofactors to increase the rate of HIV transmission and infection. Therefore, a product that inhibits HIV and HSV would have potential benefits in the prophylaxis against these sexually transmitted viruses. The labyrinthopeptin A1 (LabyA1) is a prototype peptide of a novel class of carbacyclic lantibiotics. Here, we extensively evaluated LabyA1 for its broad-spectrum activity against HIV and HSV.

## Methods

Replication of HIV-1, HIV-2 and drug (e.g. tenofovir, maraviroc, raltegravir, saquinavir)-resistant viruses were evaluated in CD4+ T cell lines and in PBMCs. LabyA1 was also tested against HSV-1 and HSV-2 and HSV-resistant viruses (such as acyclovir). It was tested also in combination with other classes of anti-HIV/HSV drugs. EC50 values and potential synergy levels were calculated using CalcuSyn software. Potential cellular side-effects, cytokine induction, toxicity and growth inhibitions were also investigated.

## Results

LabyA1 exhibited a consistent and broad anti-HIV activity (EC50: 0.70-3.3 µM) and anti-HSV activity (EC50: 0.29-2.8 µM). LabyA1 also inhibited viral cell-cell transmission between persistently HIV-infected T cells and uninfected CD4+ T cells (EC50: 2.5 µM) and inhibited the transmission of HIV captured on DC-SIGN to CD4+ T cells (EC50: 4.1 µM). LabyA1 behaves as a novel type of viral entry inhibitor. LabyA1 also demonstrated additive to synergistic effects in its anti-HIV-1 and anti-HSV-2 activity with anti(retro)viral drugs in dual combinations such as tenofovir, acyclovir, saquinavir, raltegravir and enfuvirtide. LabyA1 was equally active against all drug-resistant HIV and HSV strains. It did not induce any inflammatory cytokines/chemokines and did not affect the growth of vaginal Lactobacilli.

## Conclusions

LabyA1 has profound dual antiviral activity. Based on the lack of toxicity on the vaginal Lactobacillus strains and its synergistic/additive profile in combination with all approved anti(retro)virals, it deserves further attention as a potential microbicide candidate in the prevention of sexually transmitted (HIV/HSV) diseases.

